# Stunting and Wasting Among Indian Preschoolers have Moderate but Significant Associations with the Vegetarian Status of their Mothers

**DOI:** 10.1093/jn/nxaa042

**Published:** 2020-03-14

**Authors:** Derek D Headey, Giordano Palloni

**Affiliations:** Poverty, Health and Nutrition Division, International Food Policy Research Institute (IFPRI), Washington, DC, USA

**Keywords:** Stunting, wasting, anemia, vegetarianism, dairy

## Abstract

**Background:**

India has high rates of child undernutrition and widespread lactovegetarianism.

**Objectives:**

The objective of this study was to examine how nutrition outcomes varied among Indian preschool children in relation to the vegetarian status of their parents.

**Methods:**

The 2015–2016 National Family Health Survey (NFHS) and the 2011–2012 National Sample Survey (NSS) were used to explore associations between parental vegetarian status and child stunting and wasting at ages 0–59 mo and anemia at ages 6–59 mo. In the NFHS, self-reports on usual consumption of foods were used to classify maternal diets, whereas in the NSS lactovegetarianism was defined at the household level.

**Results:**

Compared with children of nonvegetarian mothers, children aged 24–59 mo of lactovegetarian mothers were 2.9 percentage points (95% CI: −4.0, −1.9) less likely to be stunted and children aged 6–23 mo were 1.6 points less likely to be wasted (95% CI: −3.0, −0.03), whereas children aged 6–23 mo with vegan mothers were 5.2 points more likely to be stunted (95% CI: 0.1, 9.4). When compared with nonvegetarian households, lactovegetarian households had better socioeconomic status and were more likely to consume dairy frequently. Children in nonvegetarian households consumed nondairy animal-sourced foods (ASFs) with relatively low frequency. The frequency of maternal dairy consumption was significantly associated with lower risks of child stunting and wasting.

**Conclusions:**

Anthropometric outcomes differed by maternal vegetarian status, which is itself strongly associated with socioeconomic position, location, religion, and caste.

## Introduction

Preschool Indian children have exceptionally high rates of child stunting (38.4%), wasting (18.7%), and anemia (58.4%), and India is by far the largest contributor to the global burden of these conditions (1). Since the 1990s, India's high levels of malnutrition have often been viewed as an “Asian Enigma” (2), and numerous empirical studies have sought to explain why malnutrition in India is so prevalent and persistent, including studies focusing on high levels of open defecation and enteric diseases (3), severe discrimination against girls/women (4), unusually poor maternal nutrition (5), and highly inadequate child feeding practices (6).

Surprisingly, however, scarcely any research on linear growth in India has focused on a unique feature of Indian culture, the country's exceptionally high levels of vegetarianism (typically lactovegetarianism), predominantly motivated by caste-based beliefs regarding the impurity of animal-sourced foods (ASFs). Most existing estimates suggest that just under one-third of the majority Hindu population is lactovegetarian (mostly upper castes), with smaller subsets of the population being lacto-ovovegetarian, lactopescatarian, or vegan. Vegetarianism is potentially a risk factor for various forms of malnutrition because ASFs are dense in calories, macronutrients (including high-quality protein), and multiple micronutrients such as vitamin B12 (the only dietary source), zinc, thiamin, riboflavin, choline, and vitamins A, E, B12, and D (7–9). Flesh foods are also rich in heme iron, whereas dairy contains no iron. However, dairy is somewhat unique in that it contains high concentrations of calcium as well as insulin-like growth factor-1, a hormone widely linked to linear growth (10). Bioavailability of micronutrients in ASFs is also generally much higher than in non-ASF foods, and some vegetal foods also contain antinutrients (e.g. phytates) that inhibit micronutrient absorption.

Consistent with the rich nutritional profile of ASFs, a number of experimental and observational studies have found strong positive associations between ASF consumption in early childhood and linear growth (7, 8, 11–15), although some of these found evidence of especially strong associations for dairy consumption (10, 16–18). ASF consumption among pregnant mothers may also be beneficial for a child's birth weight (15).

Cognizance of the nutritional benefits of ASF consumption for children has prompted significant international literature on the nutritional consequences of vegetarianism, mostly conducted in high-income countries. These studies raise concerns about micronutrient deficiencies as well as slower growth (19–23), although some studies found that vegetarian children are taller than nonvegetarian peers (24, 25). A study in the UK specifically focused on older children from north Indian ethnic groups in which lactovegetarian diets are common (26), although the relevance of this study to children in India is questionable given likely differences in diets and health environments.

To our knowledge, the only previous study of vegetarianism and child anthropometric outcomes in India focused on pescatarian households in fishing communities in Madras (Chennai) in the early 1980s (27). This study found that among older children in the sample (aged 36–72 mo) those in pescatarian households were taller and heavier for their age than those in households that also consumed meat. However, the relevance of this study for India as a whole is limited because most Indian vegetarians are lactovegetarian rather than pescatarian. Several studies examined vegetarianism as a risk factor for maternal and child anemia in specific geographical areas, typically cities (28–33). However, we did not find any studies that examined associations between vegetarianism and child anemia, stunting, or wasting at the national level.

Considering the knowledge gaps surrounding vegetarianism and child nutrition outcomes in India, the main objective of the present study was to explore whether different forms of vegetarianism—particularly the predominant lactovegetarian form—are risk factors for stunting, wasting, and anemia. A second objective was to explore potential explanations for any associations by examining dietary differences across vegetarian and nonvegetarian groups, as well as the associations between these dietary patterns and child nutrition outcomes.

## Methods

### Data

This study primarily used the 2015–2016 National Family Health Survey-4 (NFHS-4) from India (34) to explore the association between household lactovegetarian status and early childhood linear growth. The NFHS-4 is representative at the national level, but also at state and district levels, and contains extensive information on child nutrition outcomes, infant and young child feeding practices, a wide array of household-level variables, and parental questions on the frequency of food consumption.

We also used the 2011–2012 National Sample Survey (NSS) (35), India's main government-implemented household economic survey, to measure per capita availability of various food groups and nutrients at the household level, based on the survey's 30-d recall consumption module. The 2011–12 NSS is a large survey (100,855 households), is nationally and subnationally representative, and contains a wide array of household-level variables but does not contain any data on child nutrition outcomes or feeding practices, or on how households allocate consumption across individual members.

### Outcomes

The primary outcomes were stunting [height-for-age *z* score (HAZ) <−2], wasting [weight-for-age *z* score (WHZ) <−2], and anemia (altitude-adjusted hemoglobin <11 g/dL), with HAZ and WHZ measured against international growth standards (36). HAZ and WHZ measures excluded biologically implausible values >6 SDs from the median for their age and sex. Stunting and wasting were measured for children aged 0–59 mo, whereas anemia/hemoglobin was only measured for children aged 6–59 mo. After excluding observations with missing data for both dependent and independent variables, samples were: 222,968 children for the stunting analysis, 220,529 for wasting, and 198,848 for anemia. We also used continuous variables (HAZ, WHZ, and hemoglobin concentrations) to test the robustness of our main results.

### Dietary variables

Vegetarianism could influence a child's nutrition outcomes through the vegetarian diet of their mother (through prenatal growth mechanisms or breastmilk quantity/quality) and/or through the child's own diet. However, in this study the NFHS cannot be used to identify vegetarianism among children (due to the short 24-h recall) so we were empirically limited to using maternal vegetarianism to identify a child's exposure to both maternal vegetarianism and (potentially) their own vegetarian diet. Maternal vegetarianism can be identified because mothers were asked about usual diets (including whether they never consume a particular food), whereas for children the main caregiver is only asked whether a particular food was consumed in the previous 24 h. Clearly, 24-h recall cannot reliably be used to classify a child as vegetarian or not. Likewise, such a short recall may give misleading impressions of a child's usual diet (37). We also note that although it is possible to identify vegetarianism for fathers, dietary data for fathers was only collected for a relatively small subsample of the maternal dietary data (just 13%).

The maternal diets module asked about the frequency of consumption of different food groups, with 4 possible responses: never, occasionally, weekly, or daily. This question was asked for 7 food groups of interest: milk or curds (hereafter dairy), pulses or beans (pulses), dark green leafy vegetables, fruits, eggs, fish, and meat. The “never” category provides the basis for defining different forms of vegetarianism. Vegan mothers are defined as those who report never consuming dairy, eggs, meat, or fish. Lactovegetarian is defined as mothers that report never consuming eggs, fish, and meat, but consuming dairy occasionally, weekly, or daily. Some vegetarians in India may also consume eggs (typically in addition to dairy), so we also constructed a measure of lacto-ovovegetarian status, and some coastal communities may also consume fish (again, typically in addition to dairy) which we classify as a lactopescatarian diet.

We also used these data to construct maternal daily-equivalent consumption of each food category in order to compare maternal diets to the 24-h recall data for child diets. Specifically, mothers who reported consuming a food group daily were assigned a likelihood of 1, weekly values received a likelihood of 1/7 = 0.143, and those who reported consuming occasionally were assigned a likelihood of 1/60 = 0.016, the equivalent of consuming from that food group once every other month. The 24-h recall data on child diets collected data for slightly different food categories: legumes/nuts, vitamin A-rich fruits and vegetables, other fruits/vegetables, dairy, eggs, and a combined meat/fish category.

Our analysis of household-level NSS data could only classify different classes of vegetarianism at the household level based on reported food consumption over the past 30 d. For example, households that did not consume any nondairy ASFs in the past 30 d were classified as lactovegetarian. We also used these food consumption estimates to calculate per capita availability of various foods at the household level (corresponding to the NFHS-4 food groups) and used South Asian food composition data to convert food availability into per capita availability of various nutrients.

### Control variables

Since vegetarianism in India is predominantly the result of religious, caste, and ethnic differences, it was important for our analysis to control for an array of confounding factors that might systematically differ across India's complex social system (see **Supplemental Tables 1** and **2** for a list of the control variables used). For example, lactovegetarianism is common among upper-caste Hindus who are also likelier to be wealthier or more educated, such that controlling for these confounders to the fullest extent possible is an important aspect of our statistical analysis.

To measure household wealth in the NFHS data we constructed a wealth index using the first component of a principal components analysis applied to 42 assets and socioeconomic characteristics (see **Supplemental Table S3** for details), following longstanding conventions (38). We also employed several child, mother, and household-level characteristics as linear controls in all analyses. These are measures that are expected to be strongly correlated with either vegetarian status and/or the child-level outcomes: child sex and age in months at 6-mo intervals, maternal height, religion of the head of the household, mother's caste/tribe, maternal secondary education, access to improved drinking water, ownership of any toilet, birth order, month of interview, institutional births, and child illnesses and vaccinations. Our analysis of the 2011–12 NSS data employed a similar set of household-level control variables and district fixed effects, but child-specific indicators were not available in this dataset.

### Statistical analysis

Our statistical analysis was conducted using Stata/MP 15.1 (StataCorp). We used multivariate linear probability models for the child outcomes as a function of maternal vegetarian status (these are preferable to LOGIT/PROBIT models in the presence of fixed effects, as discussed below). We estimated clustered SEs at the district level to allow for any within-district correlation in the error terms and weighted observations using the national women's or children's weights for the mother- and child-level outcomes, respectively.

**FIGURE 1:**
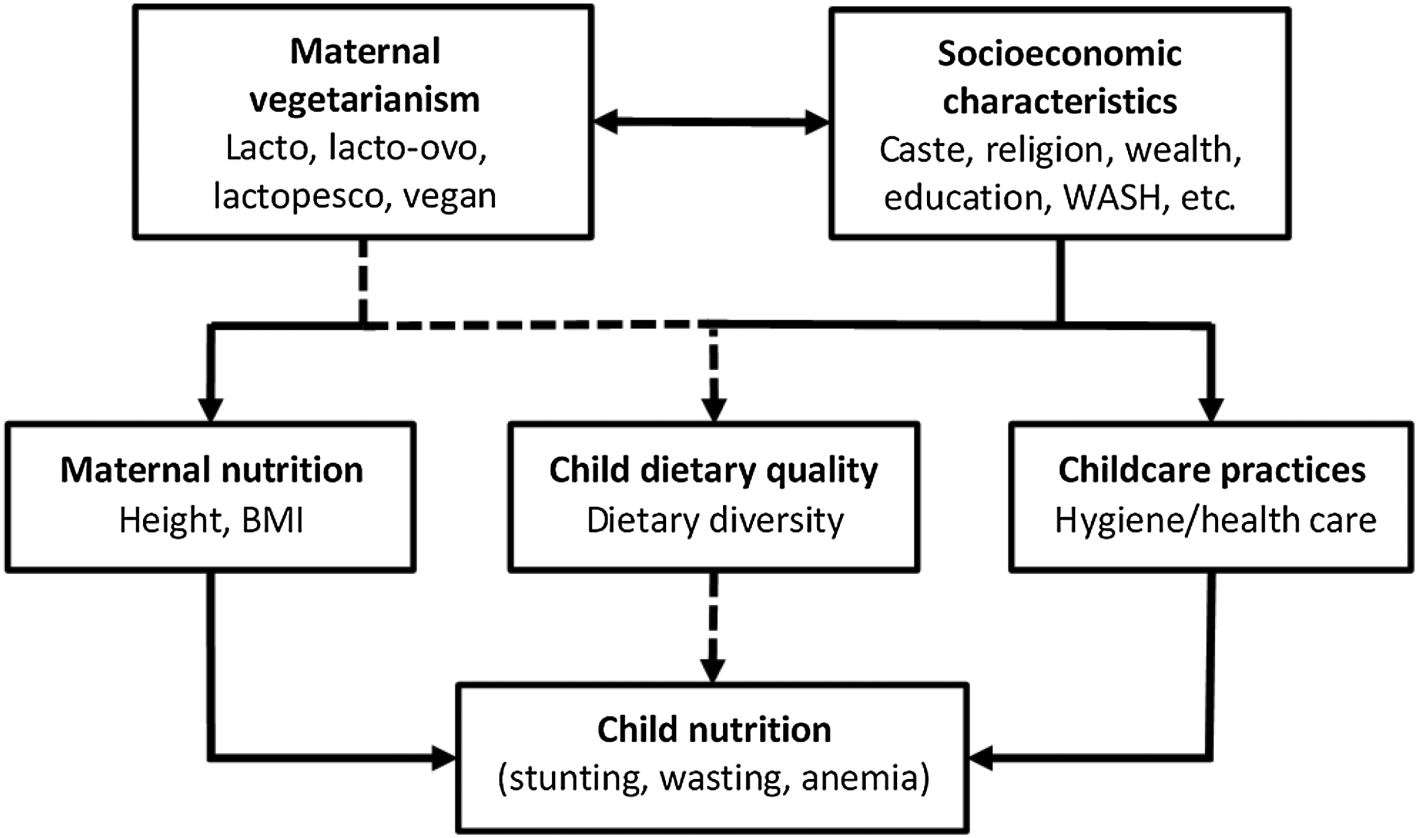
A conceptual framework for evaluating the associations between maternal vegetarian status and child nutrition outcomes. Source: authors’ construction. The dotted line pathway reflects our principal hypothesis that maternal vegetarianism is associated with child nutrition because of differences in child dietary quality between vegetarian and nonvegetarian households. WASH, Water, Sanitation and Hygiene.

Our statistical approach was guided by the conceptual framework laid out in **Figure 1**, which hypothesizes ways through which maternal vegetarian status might be associated with child nutrition outcomes. The dotted lines depict the pathway we hypothesize to be most important, with maternal vegetarianism affecting child diet quality. The solid lines depict possible confounding mechanisms: the association between vegetarianism and socioeconomic factors (notably caste/religion and wealth), maternal height or body mass, or childcare practices (e.g. hygiene, health care). We used this framework to specify the choice of control variables, but also to conduct various auxiliary tests linking dietary quality to maternal vegetarian status and child growth.

Based on Figure 1, the regression models were adjusted for the economic, religious, and demographic variables described above, whereas district fixed effects were used to absorb variation in all unobserved determinants of the outcome variables that are constant across observations within the same district. By including the district fixed effects, we restricted identifying variation to differences within a district. This strategy is particularly suitable for the Indian context, where districts are diverse with respect to child nutrition outcomes (39), and where there has been a significant push towards decentralizing policymaking decisions to the district and subdistrict levels (40).

Another important feature of our regression analysis was stratification by child age. We did this for several reasons. First, if the main mechanism linking maternal vegetarianism to child nutrition outcomes is the child's own dietary patterns—which we assumed are similar to their mother's (41)—then it is plausible that any differences in nutrition outcomes only emerge once a child is fed complementary foods from ∼6 mo onwards. Second, previous research has argued that both stunting and wasting analyses should be stratified by age, albeit for different reasons (42). As stunting is the result of an accumulation of past nutritional insults, the growth benefits of consuming a nutrient-rich diet are likely to be more apparent for older children than younger children that have only recently begun consuming nutrient-rich foods, such that samples of children aged 24–59 mo are recommended for most stunting analyses. In contrast, wasting and anemia are usually the result of more recent nutritional insults, and typically peak within the first 2 y after birth. Hence, associations with wasting and anemia are expected to be stronger for younger children (<24 mo), whereas the opposite is true for stunting (42).

Due to these considerations, we stratified the full 0–59 mo age range into 3 subsamples: *1*) a 0–5 mo period characterized by predominant breastfeeding; *2*) a 6–23 mo period characterized by the introduction of solid foods, but also high rates of wasting and anemia; and *3*) a 24–59 mo period in which children had passed the first 1000 d of life, but in which height differences between well-nourished and poorly nourished children should be more apparent. Hence, we hypothesized that any differences in child nutrition outcomes among vegetarian and nonvegetarian children would be largest in the 24–59 mo period for stunting and the 6–23 mo period for wasting and anemia. In addition to age stratification we also stratified results by wealth quintile to see whether the results only hold for poorer/wealthier populations.

To explore the possible mechanisms explaining the relations between maternal vegetarian status and child nutrition outcomes, we examined the relation between maternal vegetarian status, frequency of the mother's reported food consumption, and child nutrition outcomes. We first compared maternal and child diets for lactovegetarian and nonvegetarian subsamples, including t tests for significant differences in means across the 2 groups. We then examined differences in household availability of different foods and nutrients using the 30-d recall estimates of food consumption from the NSS using adjusted regressions analogous to those applied to the NFHS data.

We then estimated regressions for stunting and wasting against maternal daily-equivalent consumption of various food groups on the grounds that the NFHS maternal dietary data are a more reliable indicator of usual diets within the household. To further explore the importance of dairy consumption as a possible mechanism we used the adjusted regression model to test for associations between household cattle ownership and maternal vegetarian status, since ownership of cattle is often a strong predictor of more frequent milk consumption within a household (18).

Finally, we also considered nondietary pathways linking vegetarian status to child nutrition outcomes, as outlined in Figure 1. As vegetarianism in India is associated with both religious beliefs and household wealth, we considered the hypothesis that lactovegetarian households might have better hygiene status than nonvegetarian households. To explore this, we estimated adjusted regressions with ownership of an improved water source, an improved toilet, or an improved handwashing station (defined as having an observable location for handwashing with both running water and soap), and child illness indicators as dependent variables, and the usual set of maternal vegetarian diet indicators as the key explanatory variables of interest.

### Ethical considerations

All the analysis is based on publicly available and entirely deidentified data. Ethical clearance was the responsibility of the organizations conducting the fieldwork (34).

## Results

### Descriptive results

**FIGURE 2:**
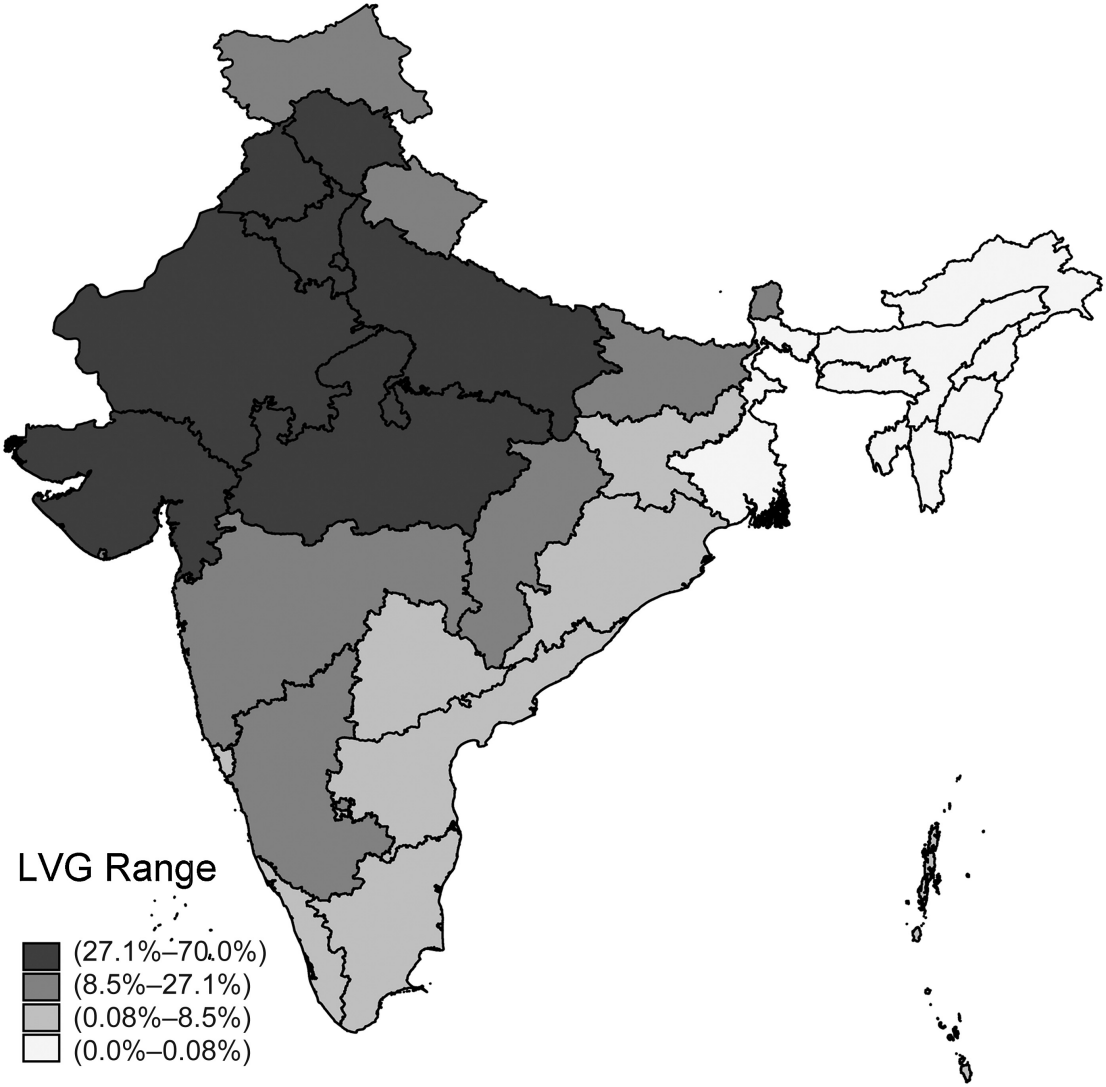
A map of the prevalence of lactovegetarianism among Indian mothers. Note: the prevalence of lactovegetarianism among Indian mothers by state. Estimated from the 2015–2016 NFHS using the women's survey weights. LVG, lactovegetarianism.

**Table 1** reports statistics on the prevalence of vegetarian and nonvegetarian diets among Indian mothers, and how this varied with social, economic, and geographic characteristics. **Figure 2** maps the prevalence of lactovegetarian mothers across Indian states (**Supplemental Figures 1–3** do the same for other categories of vegetarianism). At the national level, 22.7% of mothers in the NFHS were lactovegetarian, 3.4% were lacto-ovovegetarian, and few were lactopescatarian (0.06%) or vegan (1.1%). Among the Hindu population, 27.3% were lactovegetarian, but lactovegetarian was most prevalent among the small Jain population (89.4%), Sikhs (62.4%), and was very rare among Muslims and Christians. In the predominantly Hindu caste system, lactovegetarian prevalence was lower among the more disadvantaged scheduled castes. Lactovegetarian prevalence increased with household wealth; 14.5% of the poorest mothers were lactovegetarian, whereas 34.6% of the richest caste were lactovegetarian. Figure 2 shows that lactovegetarian status was more common in the north and north-western states and less common in the north east (a largely tribal population where ASFs are important in the diet) and in the east and south, despite majority Hindu populations there. Similar patterns held for prevalence of lacto-ovovegetarian mothers (Supplemental Figure 1).

**Table 2** reports the prevalence of stunting, wasting, and anemia in children by maternal dietary status. The reported *P* values test the null hypothesis of equality between undernutrition prevalence among children from the various maternal vegetarian groups and children of nonvegetarian mothers. Among all children aged 0–59 mo, 38.4% were stunted and 18.7% were wasted, whereas 58.4% of children aged 6–59 mo were anemic (Supplemental Table 1). There were modest but often statistically significant differences in nutrition outcomes across different dietary groups. Compared with children of nonvegetarian mothers, children of lactovegetarian mothers were significantly less likely to be stunted or wasted, but more likely to be anemic. Similar patterns held for children of lacto-ovovegetarian and lactopescatarian mothers. Children of vegan mothers were significantly more likely to be stunted, wasted, or anemic than nonvegetarian children, and the differences were relatively large (8.8 percentage points in the case of stunting).

**TABLE 1:** Prevalence of vegetarian and nonvegetarian diets among Indian mothers by location, religion, caste, and wealth^1^

		Maternal vegetarian status				
	*n* ^2^	Nonvegetarian	Lactovegetarian	Lacto-ovovegetarian	Lactopescatarian	Vegan
All households	223, 040	0.722 ± 0.448	0.227 ± 0.419	0.034 ± 0.18	0.006 ± 0.079	0.011 ± 0.106
Urban households	52,906	0.741 ± 0.438	0.209 ± 0.406	0.039 ± 0.194	0.003 ± 0.054	0.009 ± 0.092
Rural households	170,134	0.715 ± 0.451	0.234 ± 0.423	0.031 ± 0.174	0.008 ± 0.087	0.012 ± 0.111
Hindu mother	161,610	0.668 ± 0.471	0.273 ± 0.446	0.038 ± 0.191	0.008 ± 0.087	0.014 ± 0.116
Muslim mother	34,988	0.972 ± 0.165	0.014 ± 0.116	0.012 ± 0.107	0.001 ± 0.036	0.002 ± 0.039
Christian mother	17,727	0.982 ± 0.131	0.008 ± 0.088	0.005 ± 0.074	0.003 ± 0.057	0.001 ± 0.033
Sikh mother	3669	0.247 ± 0.431	0.624 ± 0.484	0.105 ± 0.307	0.001 ± 0.031	0.023 ± 0.149
Buddhist mother	2103	0.921 ± 0.269	0.038 ± 0.19	0.030 ± 0.17	0.000 ± 0.008	0.011 ± 0.105
Jain mother	188	0.052 ± 0.222	0.894 ± 0.309	0.023 ± 0.15	0.000 ± 0.000	0.031 ± 0.175
Other Religion mother	2755	0.947 ± 0.223	0.041 ± 0.199	0.003 ± 0.059	0.004 ± 0.06	0.004 ± 0.065
Scheduled caste	42,222	0.787 ± 0.409	0.159 ± 0.366	0.037 ± 0.19	0.005 ± 0.071	0.011 ± 0.105
Scheduled tribe	43,997	0.824 ± 0.381	0.132 ± 0.339	0.021 ± 0.144	0.007 ± 0.081	0.016 ± 0.124
Other backwards caste	88,115	0.680 ± 0.466	0.264 ± 0.441	0.036 ± 0.187	0.008 ± 0.087	0.012 ± 0.109
Other caste	39, 011	0.648 ± 0.478	0.302 ± 0.459	0.035 ± 0.184	0.005 ± 0.071	0.010 ± 0.097
No caste	9695	0.912 ± 0.283	0.070 ± 0.255	0.009 ± 0.094	0.003 ± 0.053	0.006 ± 0.078
Poorest quintile	54,791	0.810 ± 0.392	0.145 ± 0.353	0.018 ± 0.132	0.013 ± 0.111	0.014 ± 0.117
2nd quintile	49,460	0.743 ± 0.437	0.205 ± 0.403	0.031 ± 0.174	0.008 ± 0.087	0.013 ± 0.115
3rd quintile	43,696	0.728 ± 0.445	0.222 ± 0.416	0.034 ± 0.18	0.004 ± 0.065	0.012 ± 0.108
4th quintile	39,927	0.709 ± 0.454	0.241 ± 0.428	0.037 ± 0.189	0.004 ± 0.061	0.009 ± 0.094
Richest quintile	35,166	0.591 ± 0.492	0.346 ± 0.476	0.052 ± 0.223	0.002 ± 0.044	0.008 ± 0.09

1 Authors’ estimates of means and SDs (mean ± SD) for key variables from the NFHS 2015–2016 data by maternal vegetarian type or nonvegetarianism. All statistics use NFHS survey weights.

2 *n* denotes the number of observations with nonmissing data.

NFHS, National Family Health Survey.

**TABLE 2:** Child nutrition outcomes by maternal vegetarian status, with tests for significant differences relative to children of nonvegetarian mothers^1^

		Nonvegetarian mother	Lactovegetarian mother	Lacto-ovovegetarian mother	Lactopescatarian mother	Vegan mother
Stunted (*n* = 223, 040)	Mean (95% CI)	0.389 (0.385, 0.392)	0.367 (0.362, 0.372)	0.373 (0.359, 0.387)	0.434 (0.400, 0.467)	0.455 (0.432, 0.478)
	*P* value^2^		<0.001	0.034	0.009	<0.001
Wasted (*n* = 220,600)	Mean (95% CI)	0.189 (0.186, 0.192)	0.182 (0.177, 0.186)	0.178 (0.167, 0.189)	0.187 (0.159, 0.215)	0.215 (0.196, 0.234)
	*P* value		0.004	0.058	0.872	0.009
Anemic (*n* = 198,908)	Mean (95% CI)	0.578 (0.574, 0.581)	0.599 (0.593, 0.605)	0.604 (0.589, 0.619)	0.619 (0.583, 0.654)	0.638 (0.615, 0.662)
	*P* value		<0.001	0.001	0.023	<0.001

1 Authors’ estimates of means and 95% CIs based on the 2015–2016 NFHS data. All statistics use NFHS survey weights.

2 *P* values are from tests of the null hypothesis of equality between malnutrition prevalence among children of different varieties of vegetarian mothers and children of nonvegetarian mothers.

NFHS, National Family Health Survey.

### Vegetarianism and child nutrition outcomes

**Table 3** reports linear probability model regressions of the associations between stunting and maternal vegetarian status relative to children of nonvegetarian mothers. Children aged 0–59 mo of lactovegetarian mothers were 2.3 points less likely to be stunted than those of nonvegetarian mothers. This relation was not significant among children aged 0–5 mo (who were predominantly breastfed), significant but modest among children aged 6–23 mo, and significant and larger among children aged 24–59 mo. Similarly, children of lacto-ovovegetarian mothers were 2.1 points less likely to be stunted in the older 24–59 mo group, but there were no significant associations for children of lactopescatarian mothers. In contrast, children of vegan mothers were 5.2 points more likely to be stunted in the 6–23 mo age group, whereas the associations for the other age ranges were not statistically significant.

**TABLE 3:** Adjusted linear probability model regressions to test associations between child stunting and maternal vegetarian status relative to children of nonvegetarian mothers, stratified by age^1^

	Age range			
	0–59 mo	0–5 mo	6–23 mo	24–59 mo
Lactovegetarian	−0.023*** (−0.031, −0.014)	−0.008 (−0.033, 0.018)	−0.016* (−0.030, −0.002)	−0.029*** (−0.040, −0.019)
Lacto-ovovegetarian	−0.012 (−0.028, 0.003)	−0.028 (−0.072, 0.016)	0.004 (−0.022, 0.031)	−0.021* (−0.040, −0.003)
Lactopescatarian	−0.012 (−0.040, 0.016)	0.055 (−0.041, 0.150)	−0.023 (−0.076, 0.031)	−0.011 (−0.048, 0.026)
Vegan	0.019 (−0.005, 0.043)	0.030 (−0.071, 0.131)	0.052* (0.009, 0.094)	−0.002 (−0.033, 0.030)
*R^2^*	0.124	0.091	0.135	0.126
*n*	222,968	18,728	67,608	136,632

1 Values are βs with 95% CIs based on robust SEs clustered at the district level shown in parentheses alongside each β. All regressions use NFHS weights. Regressions are adjusted linear probability models of stunting (HAZ <−2) against the 4 categories of maternal vegetarian diets with children of nonvegetarian mothers as the omitted base category, adjusting for the control variables and fixed effects listed in the Methods section. ^#^*P* value <0.10; *P value < 0.05; **P value < 0.01; ***P value < 0.001.

NFHS, National Family Health Survey.

**Table 4** presents analogous results for wasting. Children aged 0–59 mo of lactovegetarian mothers were 0.8 points less likely to be wasted, but this result was driven by children aged 6–23 mo, who were 1.6 points less likely to be wasted. Similarly, children of lacto-ovovegetarian mothers were 2.3 points less likely to be wasted in the 6–23 mo range, but there were no significant differences in other age ranges. Relative to nonvegetarian mothers, there were no significant differences for the smaller groups of children of lactopescatarian mothers or children of vegan mothers.

**TABLE 4:** Adjusted linear probability model regressions to test associations between child wasting and maternal vegetarian status relative to children of nonvegetarian mothers, stratified by age^1^

	Age range			
	0–59 mo	0–5 mo	6–23 mo	24–59 mo
Lactovegetarian	−0.008* (−0.016, −0.000)	0.002 (−0.023, 0.028)	−0.016* (−0.030, −0.003)	−0.005 (−0.014, 0.003)
Lacto-ovovegetarian	−0.006 (−0.018, 0.007)	0.016 (−0.021, 0.053)	−0.023^#^ (−0.050, 0.004)	0.000 (−0.016, 0.015)
Lactopescatarian	−0.002 (−0.028, 0.023)	−0.023 (−0.086, 0.040)	−0.006 (−0.049, 0.037)	0.001 (−0.031, 0.034)
Vegan	0.003 (−0.016, 0.022)	0.021 (−0.034, 0.077)	0.015 (−0.031, 0.062)	−0.004 (−0.026, 0.018)
*R^2^*	0.041	0.067	0.044	0.042
*n*	220,529	17,751	66,579	136,199

1 Values are βs with 95% CIs based on robust SEs clustered at the district level shown in parentheses alongside each β. All regressions use NFHS weights. Regressions are adjusted linear probability models of wasting (WHZ <−2) against the 4 categories of maternal vegetarian diets with children of nonvegetarian mothers as the omitted base category, adjusting for the control variables and fixed effects listed in the Methods section. ^#^*P* value < 0.10; *P value < 0.05; **P value < 0.01; ***P value < 0.001.

NFHS, National Family Health Survey.

**Supplemental Table 4** reports the results for anemia. In contrast to the unadjusted differences in means reported in Table 2, lactovegetarian children were slightly less likely to be anemic than nonvegetarian children after adjusting for confounders, especially in the 6–23 mo range. However, there were no consistent associations with anemia for children of mothers following other forms of vegetarian practices.

**Supplemental Tables 5–7** show the analogous results for HAZ and WHZ, as well as for hemoglobin (g/dL). In all 3 tables, the results are consistent with the main findings in Tables 3 and 4, and Supplemental Table 4.

### Exploring dietary explanations of the association between maternal lactovegetarian status and reduced risks of stunting and wasting

To explore the scope for dietary differences to explain the lower risk of stunting and wasting among children of lactovegetarian mothers we used NFHS and NSS data to examine maternal, child, and household dietary differences, and then explored the associations between maternal/child diets and child nutrition outcomes in NFHS data.

**Table 5** reports the differences in dietary patterns between lactovegetarian mothers/children and nonvegetarian mothers/children in the NFHS using the daily-equivalence measure of consumption for mothers and the 24-h recalled consumption measure for children. Differences across the 2 groups are also reported, along with the statistical significance based on t tests of whether the child or maternal daily-equivalent consumption is the same. By definition, meat, eggs, and fish are not consumed by lactovegetarian mothers, so the consumption differences relative to nonvegetarian mothers are particularly large for these foods. However, for other food groups there were also some statistically significant differences. By far the largest difference is in dairy consumption: lactovegetarian mothers were 19.9 points more likely to consume dairy on a daily (equivalent) basis than nonvegetarian mothers, and the difference is highly statistically significant. Interestingly, lactovegetarian mothers consumed legumes and green leafy vegetables with less frequency.

**TABLE 5:** Maternal and child dietary patterns for lactovegetarian and nonvegetarian households^1^

	Maternal consumption^2^				Child consumption^3^		
	Lactovegetarian (*n* = 29,147)	Nonvegetarian (*n* = 89,580)	Difference^4^		Lactovegetarian (*n* = 19,149)	Nonvegetarian (*n* = 61,762)	Difference
Green leafy vegetables	0.465 ± 0.443	0.553 ± 0.446	−0.088***	Starches	0.655 ± 0.475	0.682 ± 0.466	−0.027***
Fruit	0.171 ± 0.307	0.168 ± 0.302	0.003	Vit. A-rich fruit & vegetables	0.353 ± 0.478	0.430 ± 0.495	−0.077***
Legumes/nuts	0.497 ± 0.436	0.523 ± 0.440	−0.026***	Other fruit & vegetables	0.212 ± 0.409	0.256 ± 0.436	−0.044***
Dairy	0.612 ± 0.455	0.413 ± 0.456	0.199***	Legumes/nuts	0.094 ± 0.292	0.153 ± 0.360	−0.059***
Eggs	0.000 ± 0.000	0.134 ± 0.217	−0.134***	Dairy	0.573 ± 0.495	0.466 ± 0.499	0.106***
Meat	0.000 ± 0.000	0.086 ± 0.127	−0.086***	Eggs	0.014 ± 0.116	0.195 ± 0.396	−0.182***
Fish	0.000 ± 0.000	0.140 ± 0.252	−0.140***	Meat/flesh foods	0.015 ± 0.123	0.142 ± 0.349	−0.126***

1 Values in the 2 leftmost columns of each panel (Maternal consumption, Child consumption) are means ± SD based on estimates from the 2015–2016 National Family Health Survey in India. All statistics use NHFS survey weights. Sample is limited to children aged 12–59 mo and mothers of children aged 12–59 mo from the main empirical sample. Only 1 observation per mother is used in the Maternal consumption panel. ^#^*P* value < 0.10; *P value < 0.05; **P value < 0.01; ***P value < 0.001.

2 Maternal consumption is based on self-reports on typical diets. Maternal daily-equivalent values are constructed by assigning a daily probability of consumption of 1 to mothers who report consuming a food group daily, a probability of 1/7 to mothers who report consuming a food group weekly, and a probability of 1/60 (once every other month) to mothers who report consuming a food group occasionally.

3 Child consumption is based on maternal reports on child consumption from each food group in the 24 h preceding the survey.

4 Differences and corresponding *P* values are from a t test of the null hypothesis of no difference in means between lactovegetarian mothers and nonvegetarian mothers.

LVG, Lactovegetarian; Non-Veg., nonvegetarian.

Compared with children of nonvegetarian mothers, we observed that lactovegetarian children were significantly more likely to have consumed dairy products in the past 24 h (10.6 points) but were again less likely to have consumed legumes/nuts, vitamin A-rich fruits/vegetables, or other fruits/vegetables. Unsurprisingly, just 1.4% of children of lactovegetarian mothers consumed eggs and 1.5% consumed meat/fish in the past 24 h. Notably though, consumption of these products in the past 24 h was also relatively low among nonvegetarian children (19.5% for eggs, 14.2% for meat/fish). Hence, although dietary diversity was generally lower for lactovegetarian mothers and their children, they were much more likely to consume dairy on a frequent basis.

**FIGURE 3:**
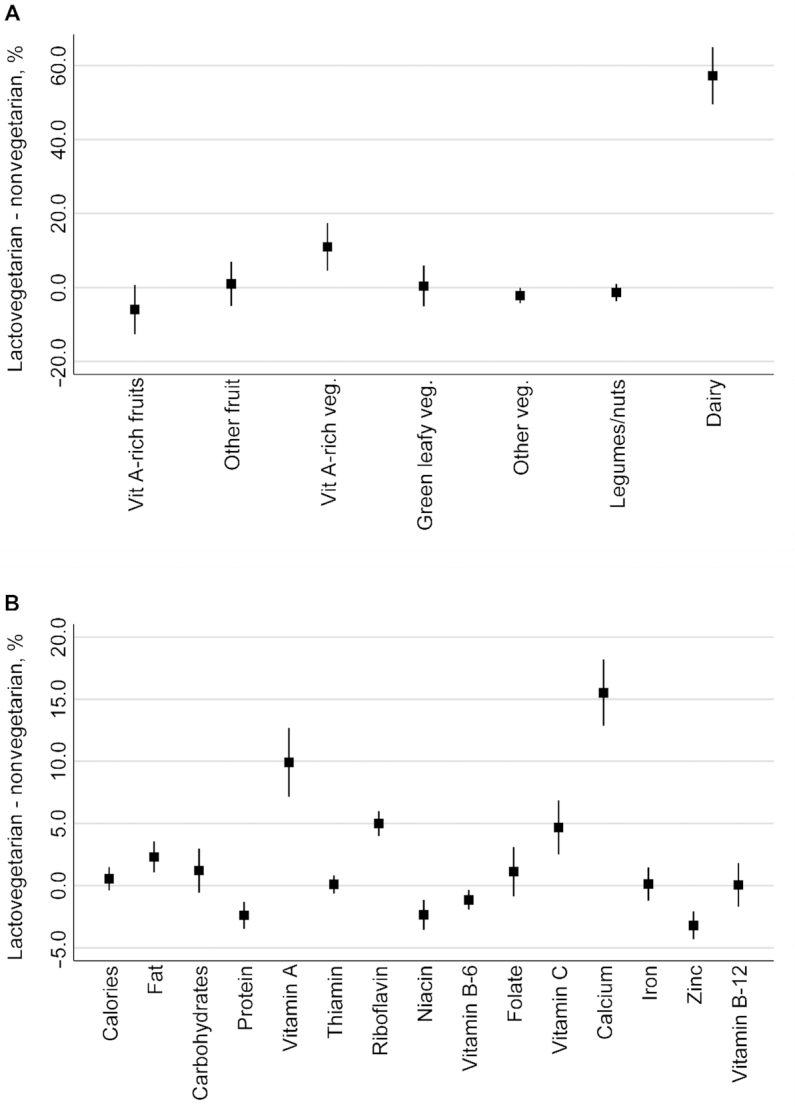
Adjusted regression-based estimates of the percentage differences in per capita availability of foods (panel A) and nutrients (panel B) between lactovegetarian and nonvegetarian households (*n =* 100,855 households). Panel A: percentage differences in the per capita availability of foods at the household level. Panel B: differences in the per capita availability of nutrients at the household level. Notes: the graphs above denote an approximation of the percentage difference between per capita supply of different foods and nutrients at the household level based on least squares regressions of the log of each food (grams) and nutrient (various units) against dummy variables for household vegetarian status, after adjusting for household characteristics. Each estimate is the result of an individual regression of each food/nutrient after applying the inverse hyperbolic sine transformation to derive an approximation of percentage differences. See Supplemental Table S2 for details of the various control variables used in these regressions.

**Figure 3** reports results from a series of adjusted regressions that use the 2011–2012 NSS data to test for significant differences per capita food availability (panel A) and nutrient availability (panel B) between lactovegetarian and nonvegetarian households. Panel A suggests that lactovegetarian households have 57.2% (95% CI: 49.5%, 65.0%) more dairy products available for consumption, as well as significantly more vitamin A-rich vegetables. Consistent with the results in panel A, lactovegetarian households have significantly and substantially more calcium available (15.5%, 95% CI: 0.128, 0.182), as well as significantly larger quantities of vitamin A, riboflavin, zinc, and fat. However, they also have moderately lower quantities of protein, niacin, vitamin B6, and zinc. Other differences were not significant, including vitamin B12, which is present in both dairy and nondairy ASFs in high quantities.

**Table 6** examines the association between the usual diet measures of maternal food group consumption and child stunting, stratified again by child age. A notable result is that only dairy consumption had a robust negative association with stunting risk. In the 24–59 mo sample, for example, daily consumption of dairy predicted a 2.2 percentage point reduction in stunting relative to children of mothers with no dairy consumption. Among other foods, pulse consumption was statistically significantly and negatively associated with stunting for children in the 0–59 mo, 6–23 mo, and 24–59 mo samples, but positively associated with stunting in the 0–5 mo sample. Fruit consumption was negatively associated with stunting in the 6–23 mo sample. It is also notable that—unlike dairy—egg, meat, and fish consumption were never significantly negatively associated with stunting reduction.

**TABLE 6:** Associations between stunting and daily-equivalent maternal consumption of various foods, stratified by child age^1^

	Age range			
	0–59 mo	0–5 mo	6–23 mo	24–59 mo
	(1)	(2)	(3)	(4)
Pulses	−0.008^#^ (−0.017, 0.001)	0.023^#^ (−0.001, 0.048)	−0.013^#^ (−0.027, 0.002)	−0.01^#^ (−0.021, 0.001)
Green leafy veg.	0.000 (−0.008, 0.009)	−0.019 (−0.042,0.005)	0.004 (−0.010, 0.019)	0.000 (−0.010, 0.011)
Fruits	−0.013^#^ (−0.028, 0.002)	0.007 (−0.030, 0.043)	−0.023* (−0.043, −0.002)	−0.013 (−0.033, 0.008)
Dairy	−0.019*** (−0.026, −0.012)	−0.027** (−0.047, −0.008)	−0.013* (−0.026, −0.001)	−0.022*** (−0.030, −0.013)
Eggs	0.008 (−0.011, 0.027)	−0.026 (−0.068, 0.016)	−0.014 (−0.043, 0.016)	0.026^#^ (−0.003, 0.055)
Fish	−0.003 (−0.025, 0.018)	−0.005 (−0.069, 0.058)	0.011 (−0.024, 0.046)	−0.008 (−0.036, 0.020)
Meat	0.026 (−0.007, 0.058)	0.084* (0.002, 0.166)	−0.039 (−0.087, 0.009)	0.052 (−0.001, 0.106)
*R* ^2^	0.124	0.093	0.136	0.127
*n*	222,968	18,728	67,608	136,632

1 Table presents βs with 95% CIs based on robust SEs clustered at the district level shown in parentheses from adjusted linear probability models regressing stunting against maternal daily-equivalent consumption of the 7 food groups listed, adjusting for the control variables and fixed effects listed in the Methods section. ^#^*P* value < 0.10; *P value < 0.05; **P value < 0.01; ***P value < 0.001. Green leafy veg., green leafy vegetables.

**Supplemental Table 8** reports analogous regressions for stunting and the consumption of 7 different food groups in the past 24 h by children aged 6–23 mo. In contrast to the results in Table 6, only a child's consumption of flesh foods predicts a reduction in stunting risk for children. However, the short 24-h recall period may induce significant measurement error into these estimates, leading to attenuation bias.

**Table 7** reports tests of the association between maternal diets and wasting, again stratified by age. Maternal dairy consumption was associated with a reduced risk of wasting in all age groups except the 0–5 mo group (when most children were breastfed). For the 6–23 mo age range, in which wasting is most prevalent, daily-equivalent dairy consumption predicted a 1.2 point reduction in wasting. For other age groups and foods there were few consistent results, although consumption of dark green leafy vegetables predicted a reduction in wasting in the 0–59 mo and 24–59 mo ranges, whereas fruit consumption predicted a 3-point reduction in wasting in the 6–23 mo range. The consumption of nondairy ASFs was never associated with a lower risk of wasting. Results for the association between wasting and children's consumption of various foods in the past 24 h are reported in **Supplemental Table 9**, but there are no robust relations between children's consumption of any food group in the past 24 h and child wasting.

**TABLE 7:** Associations between wasting and consumption of daily-equivalent consumption of various foods among mothers, stratified by child age^1^

	Age range			
	0–59 mo	0–5 mo	6–23 mo	24–59 mo
	(1)	(2)	(3)	(4)
Pulses	0.004 (−0.003, 0.011)	0.011 (−0.006, 0.029)	0.005 (−0.008, 0.017)	0.002 (−0.007, 0.011)
Grean leafy veg.	−0.009* (−0.017, −0.002)	−0.004 (−0.022,0.015)	0.001 (−0.014, 0.017)	−0.014** (−0.024, −0.005)
Fruits	−0.004 (−0.016, 0.009)	0.009 (−0.018, 0.037)	−0.03* (−0.055, −0.005)	0.008 (−0.005, 0.021)
Dairy	−0.010** (−0.016, −0.004)	−0.006 (−0.022, 0.010)	−0.012* (−0.023, −0.001)	−0.009* (−0.017, −0.002)
Eggs	0.004 (−0.011, 0.020)	0.021 (−0.019, 0.060)	0.006 (−0.025, 0.036)	0.002 (−0.017, 0.021)
Fish	−0.013 (−0.038, 0.012)	0 (−0.050, 0.050)	−0.011 (−0.054, 0.032)	−0.011 (−0.039, 0.018)
Meat	0.007 (−0.029, 0.043)	0.025 (−0.060, 0.110)	0.03 (−0.025, 0.086)	−0.013 (−0.053, 0.027)
*R* ^2^	0.041	0.067	0.044	0.042
*n*	220,529	17,751	66,579	136,199

1 Table presents βs with 95% CIs based on robust SEs clustered at the district level shown in parentheses from adjusted linear probability models regressing wasting against maternal daily-equivalent consumption of the 7 food groups listed, adjusting for the control variables and fixed effects listed in the Methods section. ^#^*P* value < 0.10; *P value < 0.05; **P value < 0.01; ***P value < 0.001. Green leafy veg., green leafy vegetables.

To further explore the hypothesis that lactovegetarian households may consume dairy more frequently or in high quantities, we also tested the association between household cattle ownership and milk consumption, since a household's own consumption of dairy products is common in developing countries because fresh milk is highly perishable (column 1 of **Supplemental Table 10**). Adjusted regressions suggested that lactovegetarian households were 13.7 points (95% CI: 12.4, 14.9) more likely to own cattle.

### Exploring the relation between hygiene indicators and vegetarian status

To test the hypothesis that vegetarians may have stronger hygiene norms (27) we used adjusted regressions to examine whether vegetarians were more likely to own improved toilets, improved drinking water, or to have improved handwashing stations with water and soap. Results are reported in columns 2–4 of Supplemental Table 10. We found some evidence that lactovegetarian mothers were significantly more likely than nonvegetarian mothers to reside in households with improved toilets and improved handwashing stations, although the size of these associations was relatively modest: 2.5 points in the case of improved toilets and 1.5 points for improved handwashing stations. We also considered child diarrhea incidence in the past 2 wk as a potential outcome of vegetarian diets (**Supplemental Table 11**). We found some evidence that children of vegan mothers are more likely to experience diarrhea, whereas children of lactovegetarian mothers were also more likely to have experienced diarrhea in the 0–5 mo range. However, in both sets of results the magnitude of these associations is consistently quite small.

### Exploring the relation between vegetarian status and child stunting by wealth quintile

To further explore the concern that vegetarians are simply wealthier than nonvegetarians we repeated our main stunting specifications for subsamples pertaining to each of 5 wealth quintiles (**Supplemental Figure 4**). Lactovegetarianism is significantly and negatively associated with stunting in each of the 5 quintiles, suggesting that the main results are unlikely to be substantially biased by the simultaneous associations between stunting, wealth, and lactovegetarianism.

## Discussion

Given that ASFs are rich in calories, high-quality protein, and a range of critical micronutrients, exposure to a strictly vegetarian diet in early childhood—or in utero via maternal nutrition—could be a significant risk factor for malnutrition in India. However, vegetarian diets are also diverse, and our results suggest that children of lactovegetarian mothers are somewhat less likely to be stunted or wasted and—in adjusted regressions—they are not significantly more likely to be anemic. In contrast, children of vegan mothers, who constitute just 1.1% of sampled Indian mothers, are at a substantially increased risk of stunting in the 6–23 mo age range.

We explored several hypotheses that might explain these results.

First, we found that lactovegetarian mothers and their children are significantly more likely to consume dairy products frequently and potentially in larger quantities, and that more frequent dairy consumption by mothers predicted a significantly lower risk of stunting and wasting in their children. This suggestive link between more frequent dairy consumption and child growth is highly consistent with observational evidence linking milk production, cow ownership, and/or dairy consumption to child growth (11, 13, 14, 18, 43), as well as a small experimental literature offering dairy supplements to young children (10). The special role of dairy products in promoting linear growth could be attributable to a number of factors, including high concentrations of vitamin B12 (44), riboflavin (45), as well as high concentrations of minerals and micronutrients (phosphorus, zinc), lactose, and the stimulating impacts of insulin-like growth factor-1 (10, 46). Another important potential explanation of the link between dairy consumption and stunting reduction in the Indian context is the rich amino acid profile of dairy products, particularly the high lysine content (47). Traditional Indian diets dominated by rice and pulses will have inadequate lysine content, with children and adolescents at greatest risk (47), particularly those suffering from enteric disorders and chronic inflammation.

Second, since our results refer to comparisons between various forms of vegetarianism and a base case of nonvegetarian diets, it is important to consider the possibility of significant dietary deficiencies in the nonvegetarian population. We showed that nondairy ASF consumption among nonvegetarian Indian children is very low, whereas a previous study of Demographic Health Surveys from 49 countries found that nondairy ASF consumption in India is also exceptionally low by international standards (11). Given that nonvegetarian children consume less dairy than lactovegetarian children, and also consume other ASFs in small quantities or low frequency, 1 interpretation of our results is that the comparison group of nonvegetarian children would substantially benefit from a higher intake of ASFs.

The lack of an association between any form of vegetarianism and anemia was also a notable finding. However, previous studies in India have not uniformly found that vegetarian children are more likely to be anemic (although most of these studies pertain to school-age children). Studies from south India—where vegetarianism is generally less common than in the north and north-west—have found that vegetarian children are less likely to be anemic, whereas studies in the north tend to find that vegetarian children are more likely to be anemic (28–33). The etiology of anemia is complex, but a lower intake of bioavailable iron in nonvegetarian populations could be mitigated by a higher intake of riboflavin and/or vitamin B12 (44, 45).

This observational study has several limitations that preclude causal inferences. As noted above, vegetarianism in India is associated with religion and caste, geography, and wealth and it is challenging to fully control for these confounders. The analysis is also constrained by measurement issues pertaining to the identification of vegetarianism, and lack of data on individual food intakes. Finally, our study is restricted to child anthropometric outcomes, rather than specific nutrient deficiencies or other health outcomes.

Although these caveats are important, this study is the first to systematically explore the associations between different types of vegetarianism and a range of child undernutrition indicators in a nationally representative sample of Indian households. Our findings suggest the need for further research, as well as reflections on how nutrition programs and food policies may need to be adjusted to maximize impact in the presence of sizable lactovegetarian populations that have often resisted efforts to supplement children with other ASFs (48). Moreover, only half of Indian children aged 6–23 mo consumed milk in the past 24 h in the 2015–2016 NFHS, suggesting there is considerable scope for India—now the world's largest dairy producer—to further improve access to dairy products, and to more closely integrate dairy into national nutrition strategies. However, for the majority nonvegetarian population it is also essential to improve the affordability and accessibility of dairy and nondairy ASFs, and to raise caregiver knowledge on the benefits of ASFs for both prenatal and postnatal nutrition.

## Supplementary Material

nxaa042_Supplemental_Files

## Notes

This research was conducted under the “Advancing Research on Nutrition and Agriculture (AReNA) ” project, Phase 2, supported by The Bill & Melinda Gates Foundation (Investment ID: OPP1177007) and the Consultative Group on International Agricultural Research (CGIAR) Program on Agriculture for Nutrition and Health (A4NH).

Author disclosures: The authors report no conflicts of interest.

Supplemental Tables 1–11 and Supplemental Figures 1–4 are available from the “Supplementary data” link in the online posting of the article and from the same link in the online table of contents at https://academic.oup.com/jn/.

Abbreviations used: ASF, animal-sourced food; HAZ, height-for-age *z* score; NFHS, National Family Health Survey-4; NSS, National Sample Survey; WHZ, weight-for-age *z* score.
