# Supplementary material for: Stunting and Wasting Among Indian Preschoolers have Moderate but Significant Associations with the Vegetarian Status of their Mothers
Source: J Nutr. 2020 Mar 14;150(6):1579–89. doi: 10.1093/jn/nxaa042 (PMC7269725; doi:10.1093/jn/nxaa042)
Supplement: nxaa042_Supplemental_Files [file nxaa042_supplemental_files.zip › Online Supplemental Figure 1.docx]

**Children of lacto-vegetarian parents in India are less likely to be stunted or wasted than their non-vegetarian peers -** Derek D. Headey and Giordano E. Palloni

**Online Supplementary Material**

**Supplemental Figure 1. A map of the prevalence of ovo-lacto-vegetarianism among Indian mothers**


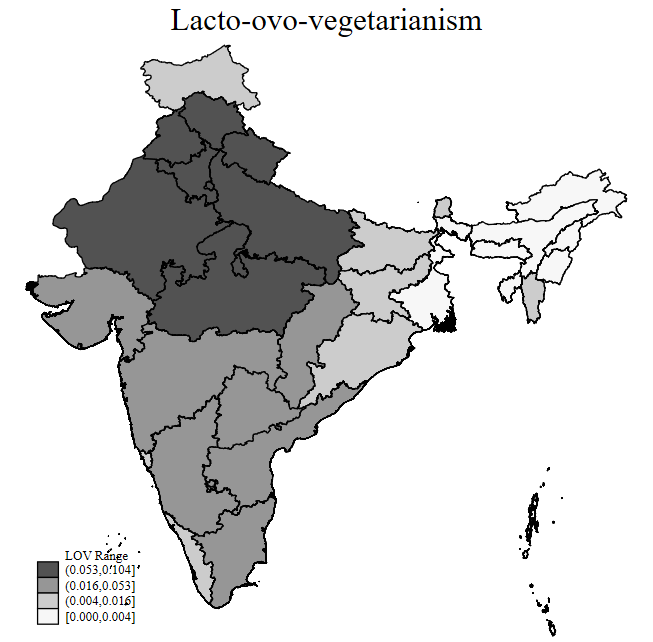


Notes: The prevalence of ovo-lacto-vegetarianism among Indian mothers by State. Estimated from the 2015-16 NHFS [34] using the women’s survey weights. Figure legend displays the range of lacto-ovo-vegetarianism prevalence by quartile. LOV, lacto-ovo-vegetarianism.
