# Supplementary material for: Stunting and Wasting Among Indian Preschoolers have Moderate but Significant Associations with the Vegetarian Status of their Mothers
Source: J Nutr. 2020 Mar 14;150(6):1579–89. doi: 10.1093/jn/nxaa042 (PMC7269725; doi:10.1093/jn/nxaa042)
Supplement: nxaa042_Supplemental_Files [file nxaa042_supplemental_files.zip › Online Supplemental Figure 3.docx]

**Supplemental Figure 3 A map of the prevalence of veganism among Indian mothers**


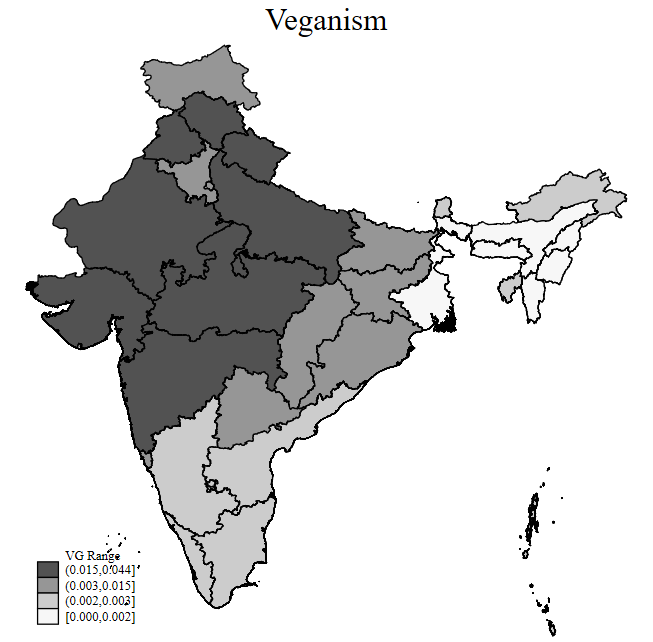


Notes: The prevalence of veganism among Indian mothers by State. Estimated from the 2015-16 NHFS [34] using the Women’s survey weights. Figure legend displays the range of veganism prevalence by quartile. VG, Veganism.
