# Supplementary material for: Stunting and Wasting Among Indian Preschoolers have Moderate but Significant Associations with the Vegetarian Status of their Mothers
Source: J Nutr. 2020 Mar 14;150(6):1579–89. doi: 10.1093/jn/nxaa042 (PMC7269725; doi:10.1093/jn/nxaa042)
Supplement: nxaa042_Supplemental_Files [file nxaa042_supplemental_files.zip › Online Supplemental Figure 4.docx]

**Supplemental Figure 4. Stunting associations with maternal lacto-vegetarianism, by household wealth quintile**


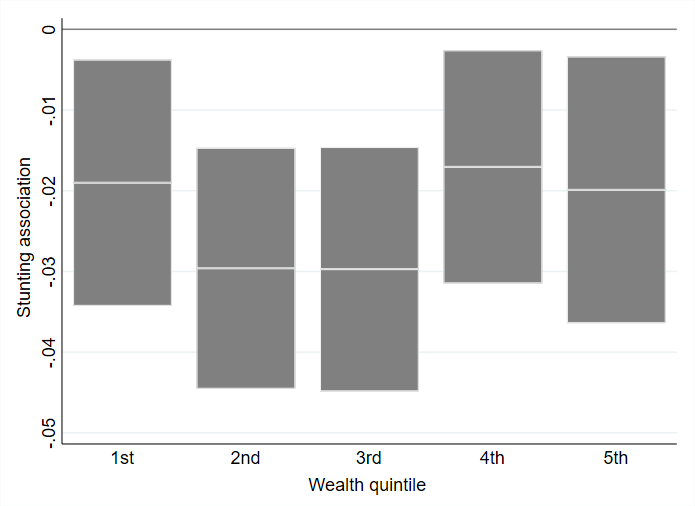


Notes: Figure displays maternal lacto-vegetarianism associations with child stunting along with 95% confidence intervals from adjusted linear probability models of stunting against the four categories of maternal vegetarian diets with children of non-vegetarian mothers as the omitted base category using data from the 2015-2016 NFHS [34]. All estimates adjust for the control variables and fixed effects listed in the Methods section and include interactions between the maternal vegetarianism indicators and household wealth quintile, with the 1^st^ quintile (*n=*54,762) containing the poorest 20% of households, the 2^nd^ quintile (*n=*49,442) containing the next poorest 20% of households, the 3^rd^ quintile (*n*=43,684) containing the middle 20% of households, the 4^th^ quintile (*n*=39,920) containing the next 20% of households, and the 5^th^ quintile (*n*=35,160) containing the wealthiest 20% of households. 95% CI based on cluster robust standard errors clustered at the district-level. All regressions use NFHS weights.
