# Supplementary material for: Stunting and Wasting Among Indian Preschoolers have Moderate but Significant Associations with the Vegetarian Status of their Mothers
Source: J Nutr. 2020 Mar 14;150(6):1579–89. doi: 10.1093/jn/nxaa042 (PMC7269725; doi:10.1093/jn/nxaa042)
Supplement: nxaa042_Supplemental_Files [file nxaa042_supplemental_files.zip › Online Supplemental Table 1.docx]

**Children of lacto-vegetarian parents in India are less likely to be stunted or wasted than their non-vegetarian peers -** Derek D. Headey and Giordano E. Palloni

**Online Supplementary Material**

**Supplemental Table 1.** Summary statistics for variables used in the study^1^

|  | Mean ±SD | *n* |
| --- | --- | --- |
| Stunted (HAZ < -2) | 0.384 ±0.486 | 223,040 |
| Height-for-Age Z-Score | -1.49 ±1.67 | 223,040 |
| Wasted (WHZ < -2) | 0.187 ±0.390 | 220,600 |
| Weight-for-Height Z-Score | -1.08 ±1.12 | 220,600 |
| Anemic (Hemoglobin < 11.0 g/dL) | 0.584 ±0.493 | 198,908 |
| Hemoglobin (g/dL) | 10.6±1.50 | 198,908 |
| Lacto-vegetarian Mother | 0.227 ±0.419 | 223,040 |
| Lacto-ovo-vegetarian Mother | 0.034 ±0.180 | 223,040 |
| Lacto-pescatarian Mother | 0.006 ±0.079 | 223,040 |
| Vegan Mother | 0.011 ±0.106 | 223,040 |
| Non-Vegetarian Mother | 0.722 ±0.448 | 223,040 |
| Head of household Christian | 0.020 ±0.141 | 223,040 |
| Head of household Hindu | 0.784 ±0.412 | 223,040 |
| Head of household Muslim | 0.168 ±0.374 | 223,040 |
| Head of household Other Religion | 0.028 ±0.165 | 223,040 |
| Mother No Caste or Tribe or Unknown | 0.042 ±0.202 | 223,040 |
| Mother Other Caste or Tribe | 0.194 ±0.396 | 223,040 |
| Mother Other Backwards Caste | 0.443 ±0.497 | 223,040 |
| Mother Scheduled Tribe | 0.103 ±0.304 | 223,040 |
| Mother Scheduled Caste | 0.217 ±0.412 | 223,040 |
| Child Age in Months | 30.1±17.0 | 223,040 |
| Male Child | 0.519 ±0.500 | 223,040 |
| PCA Based Asset Index Score | -0.166 ±2.63 | 223,040 |
| Maternal Height (cm) | 152±6.10 | 223,040 |
| Household Has Improved Drinking Water | 0.897 ±0.304 | 223,040 |
| Household Open Defecation | 0.467 ±0.499 | 223,040 |
| Mother Has Some Secondary School | 0.535 ±0.499 | 223,040 |
| Birth Order | 2.19 ±1.40 | 223,040 |
| Month of Interview | 4.69 ±1.85 | 223,040 |
| Institutional Birth | 0.791 ±0.406 | 223,040 |
| Child had any illnesses in the past 2 weeks | 0.224 ±0.417 | 222,968 |
| Child received 3 doses of polio vaccine | 0.618 ±0.486 | 223,040 |
| Child received 3 doses DPT vaccine | 0.691 ±0.462 | 223,040 |
| Child received measles vaccine | 0.683 ±0.465 | 223,040 |
| Child received BCG vaccine | 0.880 ±0.324 | 223,040 |

^1^Estimates from the 2015-2016 NFHS in India [34]. Dietary variables are defined using self-reports on typical diets. Anemia is defined using altitude-adjusted hemoglobin. All statistics use NFHS survey weights. BCG, Bacille Calmette-Guerin; DPT, Diptheria, pertussis, and tetanus; PCA, Principal Component Analysis.
