# Supplementary material for: Stunting and Wasting Among Indian Preschoolers have Moderate but Significant Associations with the Vegetarian Status of their Mothers
Source: J Nutr. 2020 Mar 14;150(6):1579–89. doi: 10.1093/jn/nxaa042 (PMC7269725; doi:10.1093/jn/nxaa042)
Supplement: nxaa042_Supplemental_Files [file nxaa042_supplemental_files.zip › Online Supplemental Table 10.docx]

**Supplemental Table 10.** Adjusted linear probability model regressions to test associations between household cattle ownership, improved water, sanitation and hygiene and maternal vegetarian status^1^

|  | Household Owns Cattle | Improved Toilet | Improved Water | Improved Handwashing |
| --- | --- | --- | --- | --- |
|  |  |  |  |  |
| Lacto-vegetarian | 0.137*** (0.124,0.149) | 0.025*** (0.017,0.033) | 0.003 (-0.005,0.011) | 0.015** (0.005,0.024) |
| Lacto-ovo-vegetarian | 0.064*** (0.046,0.082) | 0.009 (-0.004,0.021) | 0.004 (-0.007,0.015) | 0.015* (0.000,0.030) |
| Lacto-pescatarian | 0.043* (0.004,0.082) | 0.005 (-0.012,0.023) | -0.006 (-0.026,0.014) | -0.015 (-0.044,0.014) |
| Vegan | 0.022 (-0.009,0.053) | -0.013 (-0.034,0.008) | 0.000 (-0.019,0.020) | -0.025^#^ (-0.051,0.000) |
| *R*^2^ | 0.223 | 0.64 | 0.142 | 0.32 |
| *n* | 166,422 | 166,422 | 166,422 | 166,422 |

^1^Values are βs with 95% confidence intervals based on robust standard errors clustered at the district-level shown in parentheses alongside each β. All regressions use the 2015-2016 NFHS data [34] and NFHS weights. Regressions are adjusted linear probability models of the outcomes (whether the household owns cattle, whether the household has improved sanitation, whether the household has an improved water source, and whether the household has an observable location for hand washing with both running water and soap available) against the four categories of maternal vegetarian diets with children of non-vegetarian mothers as the omitted base category, adjusting for the control variables and fixed effects listed in the Methods section, but excluding the outcomes as controls when they are the dependent variable. The sample is limited to one observation per mother for the mothers of the children included in the main sample. ^#^ *P*-value < 0.10; * *P*-value < 0.05; ** *P*-value < 0.01; *** *P*-value < 0.001. LOV, Lacto-ovo-vegetarian; LPV, Lacto-pescatarian; LVG, Lacto-vegetarian; VG, Vegan.
