# Supplementary material for: Stunting and Wasting Among Indian Preschoolers have Moderate but Significant Associations with the Vegetarian Status of their Mothers
Source: J Nutr. 2020 Mar 14;150(6):1579–89. doi: 10.1093/jn/nxaa042 (PMC7269725; doi:10.1093/jn/nxaa042)
Supplement: nxaa042_Supplemental_Files [file nxaa042_supplemental_files.zip › Online Supplemental Table 11.docx]

**Supplemental Table 11.** Adjusted linear probability model regressions to test associations between child diarrhea and maternal vegetarian status relative to children of non-vegetarian mothers, stratified by age^1^

|  | Age Range | | | |
| --- | --- | --- | --- | --- |
|  | 0-59mo | 0-5mo | 6-23mo | 24-59mo |
| Lacto-vegetarian | 0.000 (-0.004,0.004) | 0.018* (0.004,0.032) | 0.002 (-0.007,0.010) | -0.003 (-0.008,0.002) |
| Lacto-ovo-vegetarian | 0.003 (-0.005,0.010) | 0.019 (-0.008,0.046) | 0.001 (-0.013,0.016) | 0.001 (-0.007,0.010) |
| Lacto-pescatarian | 0.009 (-0.007,0.025) | -0.001 (-0.056,0.055) | 0.026* (0.001,0.052) | 0.003 (-0.017,0.023) |
| Vegan | 0.022** (0.007,0.037) | -0.004 (-0.053,0.044) | 0.038* (0.008,0.068) | 0.018* (0.002,0.034) |
| *R*^2^ | 0.373 | 0.457 | 0.424 | 0.315 |
| *n* | 222,832 | 18,721 | 67,569 | 136,542 |

^1^Values are βs with 95% confidence intervals based on robust standard errors clustered at the district-level shown in parentheses alongside each β. All regressions use the 2015-2016 NFHS data [34] and NFHS weights. Regressions are adjusted linear probability models of whether children had diarrhea in the two weeks preceding the survey against the four categories of maternal vegetarian diets with children of non-vegetarian mothers as the omitted base category, adjusting for the control variables and fixed effects listed in the Methods section. ^#^ *P*-value < 0.10; * *P*-value < 0.05; ** *P*-value < 0.01; *** *P*-value < 0.001.
