# Supplementary material for: Stunting and Wasting Among Indian Preschoolers have Moderate but Significant Associations with the Vegetarian Status of their Mothers
Source: J Nutr. 2020 Mar 14;150(6):1579–89. doi: 10.1093/jn/nxaa042 (PMC7269725; doi:10.1093/jn/nxaa042)
Supplement: nxaa042_Supplemental_Files [file nxaa042_supplemental_files.zip › Online Supplemental Table 2.docx]

**Supplemental Table 2.** Summary statistics for the 2011-2012 National Sample Survey

|  | Mean ±SD | *n* |
| --- | --- | --- |
| Per capita Vitamin A (µg /day) | 148±104 | 100,977 |
| Per capita Thiamin (mg/day) | 0.627±0.290 | 100,977 |
| Per capita Riboflavin (mg/day) | 0.758±0.459 | 100,977 |
| Per capita Niacin (mg/day) | 13.3±4.85 | 100,977 |
| Per capita Vitamin B6 (mg/day) | 0.715±0.342 | 100,977 |
| Per capita Folate (mg/day) | 99.7±40.3 | 100,977 |
| Per capita Vitamin C (mg/day) | 27.2±19.6 | 100,977 |
| Per capita Calcium (mg/day) | 403±286 | 100,977 |
| Per capita Iron (mg/day) | 12.3±5.63 | 100,977 |
| Per capita Zinc (mg/day) | 7.73±2.70 | 100,977 |
| Per capita Vitamin B12 (µg /day) | 0.904±0.896 | 100,977 |
| Per capita Phenylalanie (mg/day) | 2247±746 | 100,977 |
| Per capita Valine (mg/day) | 2296±765 | 100,977 |
| Per capita Threonine (mg/day) | 1516±515 | 100,977 |
| Per capita Tryptophan (mg/day) | 488±169 | 100,977 |
| Per capita Methionine (mg/day) | 909±308 | 100,977 |
| Per capita Leucine (mg/day) | 3529±1187 | 100,977 |
| Per capita Isoleucine (mg/day) | 1699±574 | 100,977 |
| Per capita Lysine (mg/day) | 1947±753 | 100,977 |
| Per capita Histidine (mg/day) | 1078±367 | 100,977 |
| Per capita Calories | 2103±527 | 100,977 |
| Per capita legumes, nuts, and seeds (g/day) | 30.9±18.8 | 100,977 |
| Per capita Vitamin A rich fruits (g/day) | 6.86±22.3 | 100,977 |
| Per capita other fruits (g/day) | 23.9±36.6 | 100,977 |
| Per capita Vitamin A rich vegetables (g/day) | 9.48±13.0 | 100,977 |
| Per capita green leafy vegetables (g/day) | 11.8±14.6 | 100,977 |
| Per capita other vegetables (g/day) | 93.2±57.0 | 100,977 |
| Per capita fish (g/day) | 7.04±19.0 | 100,977 |
| Per capita meat and poultry (g/day) | 7.02±11.1 | 100,977 |
| Per capita eggs (g/day) | 3.11±5.67 | 100,977 |
| Per capita dairy (g/day) | 159±171 | 100,977 |
| Lactovegetarian household | 0.316±0.465 | 100,977 |
| Ovo-lactovegetarian household | 0.035±0.183 | 100,977 |
| Pescatarian household | 0.055±0.227 | 100,977 |
| Vegan household | 0.059±0.236 | 100,977 |
| Non-vegetarian household | 0.491±0.500 | 100,977 |
| Per capita total household expenditure per year (Rupee) | 17131±15798 | 100,977 |
| Male head of household | 0.881±0.324 | 100,977 |
| Head of household age | 45.3±13.8 | 100,977 |
| Head of household completed primary | 0.564±0.496 | 100,970 |
| Household has ration card | 0.801±0.399 | 100,964 |
| Household size | 4.44±2.21 | 100,977 |
| Housheold had a ceremony in past 30 days | 0.012±0.109 | 100,913 |
| Household owns land | 0.859±0.348 | 100,977 |
| Hindu household | 0.832±0.374 | 100,974 |
| Muslim household | 0.118±0.323 | 100,974 |
| Christian household | 0.024±0.155 | 100,974 |
| Household other religion | 0.026±0.158 | 100,974 |
| Scheduled tribe | 0.089±0.285 | 100,964 |
| Scheduled caste | 0.191±0.393 | 100,964 |
| Other Backwards Caste | 0.431±0.495 | 100,964 |
| Other Caste or Tribe | 0.289±0.454 | 100,964 |

Notes: Estimates of Means±SDs from the 2011-2012 National Sample Survey (NSS) in India [35]. All statistics use NSS survey weights.
