# Supplementary material for: Stunting and Wasting Among Indian Preschoolers have Moderate but Significant Associations with the Vegetarian Status of their Mothers
Source: J Nutr. 2020 Mar 14;150(6):1579–89. doi: 10.1093/jn/nxaa042 (PMC7269725; doi:10.1093/jn/nxaa042)
Supplement: nxaa042_Supplemental_Files [file nxaa042_supplemental_files.zip › Online Supplemental Table 3.docx]

**Supplemental Table 3.** Factor loadings for the first principal component in the wealth index^1^

| **Characteristic** | **Factor Loading** |
| --- | --- |
|  |  |
| Female Headed | -0.01 |
| Number of Members | 0.00 |
| Owns Mattress | 0.19 |
| Owns Pressure Cooker | 0.24 |
| Owns Chair | 0.17 |
| Owns Cot or Bed | 0.07 |
| Owns Radio | 0.04 |
| Owns TV or VCR | 0.24 |
| Owns Sewing Machine | 0.18 |
| Has Mobile Telephone | 0.12 |
| Has Landline Telephone | 0.10 |
| Has Internet | 0.16 |
| Has Computer | 0.17 |
| Has Air Conditioner | 0.21 |
| Has Washing Machine | 0.21 |
| Has Watch | 0.17 |
| Has Water Pump | 0.13 |
| Has Thresher | 0.03 |
| Has Tractor | 0.05 |
| Has Motorcycle/Scooter | 0.21 |
| Has Animal Drawn Cart | 0.00 |
| Has Car/Truck | 0.14 |
| Has Bank Account | 0.09 |
| Has BPL Card | -0.12 |
| Has Mosquito Bednet | -0.07 |
| Urban cluster | 0.21 |
| Owns Agricultural Land | -0.07 |
| Owns Cattle | -0.11 |
| Owns Camels | -0.01 |
| Owns Horse/Donkey/Mule | -0.01 |
| Owns Goat | -0.11 |
| Owns Sheep | -0.03 |
| Owns Chickens/Ducks | -0.11 |
| Member has Health Insurance | 0.01 |
| Has Electricity | 0.16 |
| Mud/clay/earth Floor | -0.28 |
| Brick/stone Floor | 0.04 |
| Wood/Tiles/Cement Floor | 0.26 |
| Leaf/Mud/Grass or No Roof | -0.10 |
| Metal/Wood Roof | -0.09 |
| Concrete/Slate Roof | 0.16 |
| Mud/Grass or No Walls | -0.24 |
| Wood/Shingles Walls | 0.24 |

^1^Eigenvectors for the first principal component of the principal component analysis (PCA) used to construct household wealth quintiles from the 2015-2016 NFHS in India [34]. The PCA restricts the data to one observation per household. BPL, Below Poverty Line.
