# Supplementary material for: Stunting and Wasting Among Indian Preschoolers have Moderate but Significant Associations with the Vegetarian Status of their Mothers
Source: J Nutr. 2020 Mar 14;150(6):1579–89. doi: 10.1093/jn/nxaa042 (PMC7269725; doi:10.1093/jn/nxaa042)
Supplement: nxaa042_Supplemental_Files [file nxaa042_supplemental_files.zip › Online Supplemental Table 4.docx]

**Supplemental Table 4.** Adjusted linear probability model regressions to test associations between child anemia and maternal vegetarian status relative to children of non-vegetarian mothers, stratified by age^1^

|  | Age Range | | |
| --- | --- | --- | --- |
|  | 6-59mo^2^ | 6-23mo | 24-59mo |
| Lacto-vegetarian | -0.007 (-0.017,0.002) | -0.014^#^ (-0.029,0.001) | -0.004 (-0.016,0.007) |
| Lacto-ovo-vegetarian | 0.004 (-0.013,0.020) | -0.012 (-0.041,0.017) | 0.011 (-0.008,0.030) |
| Lacto-pescatarian | 0.004 (-0.034,0.042) | 0.052* (0.011,0.094) | -0.018 (-0.066,0.030) |
| Vegan | 0.007 (-0.018,0.031) | 0.006 (-0.035,0.047) | 0.008 (-0.020,0.036) |
| *R^2^* | 0.117 | 0.09 | 0.101 |
| *n* | 198,848 | 64,374 | 134,474 |

^1^Values are βs with 95% confidence intervals based on robust standard errors clustered at the district-level shown in parentheses alongside each β. All regressions use the 2015-2016 NFHS data [34] and use NFHS weights. Regressions are adjusted linear probability models of anemia (altitude adjusted hemoglobin < 11 g/dL) against the four categories of maternal vegetarian diets with children of non-vegetarian mothers as the omitted base category, adjusting for the control variables and fixed effects listed in the Methods section. ^#^ *P*-value < 0.10; * *P*-value < 0.05; ** *P*-value < 0.01; *** *P*-value < 0.001.

^2^Anemia is only measured for children 6-59mo in the NFHS.
