# Supplementary material for: Stunting and Wasting Among Indian Preschoolers have Moderate but Significant Associations with the Vegetarian Status of their Mothers
Source: J Nutr. 2020 Mar 14;150(6):1579–89. doi: 10.1093/jn/nxaa042 (PMC7269725; doi:10.1093/jn/nxaa042)
Supplement: nxaa042_Supplemental_Files [file nxaa042_supplemental_files.zip › Online Supplemental Table 5.docx]

**Supplemental Table 5.** Adjusted linear probability model regressions to test associations between child Height-for-age Z-score (HAZ) and maternal vegetarian status relative to children of non-vegetarian mothers, stratified by age^1^

|  | Age Range | | | |
| --- | --- | --- | --- | --- |
|  | 0-59mo | 0-5mo | 6-23mo | 24-59mo |
| Lacto-vegetarian | 0.059** (0.025,0.092) | 0.015 (-0.113,0.144) | 0.035 (-0.025,0.094) | 0.080*** (0.044,0.117) |
| Lacto-ovo-vegetarian | -0.010 (-0.061,0.040) | -0.033 (-0.247,0.181) | -0.026 (-0.116,0.064) | 0.009 (-0.045,0.063) |
| Lacto-pescatarian | 0.039 (-0.059,0.137) | -0.207 (-0.620,0.205) | 0.167 (-0.042,0.376) | 0.001 (-0.104,0.106) |
| Vegan | -0.08^#^ (-0.162,0.003) | -0.183 (-0.511,0.146) | -0.115 (-0.264,0.035) | -0.040 (-0.151,0.070) |
| *R*^2^ | 0.163 | 0.111 | 0.151 | 0.155 |
| *n* | 222,968 | 18,728 | 67,608 | 136,632 |

^1^Values are βs with 95% confidence intervals based on robust standard errors clustered at the district-level shown in parentheses alongside each β. All regressions use 2015-2016 NFHS data [34] and NFHS weights. Regressions are from adjusted linear probability models of Height-for-age Z-score (HAZ) against the four categories of maternal vegetarian diets with children of non-vegetarian mothers as the omitted base category, adjusting for the control variables and fixed effects listed in the Methods section. ^#^ *P*-value < 0.10; * *P*-value < 0.05; ** *P*-value < 0.01; *** *P*-value < 0.001.
