# Supplementary material for: Stunting and Wasting Among Indian Preschoolers have Moderate but Significant Associations with the Vegetarian Status of their Mothers
Source: J Nutr. 2020 Mar 14;150(6):1579–89. doi: 10.1093/jn/nxaa042 (PMC7269725; doi:10.1093/jn/nxaa042)
Supplement: nxaa042_Supplemental_Files [file nxaa042_supplemental_files.zip › Online Supplemental Table 6.docx]

**Supplemental Table 6.** Adjusted linear probability model regressions to test associations between child Weight-for-height Z-score (WHZ) and maternal vegetarian status relative to children of non-vegetarian mothers, stratified by age^1^

|  | Age Range | | | |
| --- | --- | --- | --- | --- |
|  | 0-59mo | 0-5mo | 6-23mo | 24-59mo |
| Lacto-vegetarian | 0.025^#^ (-0.004,0.053) | 0.006 (-0.119,0.132) | 0.059* (0.014,0.104) | 0.011 (-0.021,0.044) |
| Lacto-ovo-vegetarian | -0.004 (-0.050,0.042) | -0.085 (-0.264,0.094) | 0.041 (-0.055,0.137) | -0.018 (-0.066,0.031) |
| Lacto-pescatarian | 0.007 (-0.067,0.082) | -0.056 (-0.537,0.424) | -0.028 (-0.177,0.121) | 0.032 (-0.054,0.119) |
| Vegan | 0.001 (-0.068,0.070) | -0.100 (-0.379,0.180) | -0.019 (-0.156,0.118) | 0.017 (-0.062,0.097) |
| *R*^2^ | 0.051 | 0.09 | 0.074 | 0.066 |
| *n* | 222,967 | 18,728 | 67,608 | 136,631 |

^1^Values are βs with 95% confidence intervals based on robust standard errors clustered at the district-level shown in parentheses alongside each β. All regressions use the 2015-2016 NFHS data [34] and use NFHS weights. Regressions are from adjusted linear probability models of Weight-for-height Z-score (WHZ) against the four categories of maternal vegetarian diets with children of non-vegetarian mothers as the omitted base category, adjusting for the control variables and fixed effects listed in the Methods section. ^#^ *P*-value < 0.10; * *P*-value < 0.05; ** *P*-value < 0.01; *** *P*-value < 0.001.
