# Supplementary material for: Stunting and Wasting Among Indian Preschoolers have Moderate but Significant Associations with the Vegetarian Status of their Mothers
Source: J Nutr. 2020 Mar 14;150(6):1579–89. doi: 10.1093/jn/nxaa042 (PMC7269725; doi:10.1093/jn/nxaa042)
Supplement: nxaa042_Supplemental_Files [file nxaa042_supplemental_files.zip › Online Supplemental Table 7.docx]

**Supplemental Table 7.** Adjusted linear probability model regressions to test associations between child hemoglobin (g/dL) and maternal vegetarian status relative to children of non-vegetarian mothers, stratified by age^1^

|  | Age Range | | |
| --- | --- | --- | --- |
|  | 6-59mo^2^ | 6-23mo | 24-59mo |
| Lacto-vegetarian | 0.027^#^ (-0.004,0.058) | 0.015 (-0.039,0.068) | 0.032^#^ (-0.001,0.065) |
| Lacto-ovo-vegetarian | -0.017 (-0.067,0.033) | 0.015 (-0.088,0.118) | -0.035 (-0.089,0.019) |
| Lacto-pescatarian | 0.054 (-0.041,0.150) | -0.015 (-0.168,0.138) | 0.081 (-0.043,0.206) |
| Vegan | -0.048 (-0.125,0.029) | -0.086 (-0.232,0.059) | -0.036 (-0.117,0.046) |
| *R*^2^ | 0.155 | 0.125 | 0.134 |
| *n* | 198,848 | 64,374 | 134,474 |

^1^Values are βs with 95% confidence intervals based on robust standard errors clustered at the district-level shown in parentheses alongside each β. All regressions use the 2015-2016 NFHS data [34] and NFHS weights. Regressions are adjusted linear probability models of hemoglobin (altitude adjusted in g/dL) against the four categories of maternal vegetarian diets with children of non-vegetarian mothers as the omitted base category, adjusting for the control variables and fixed effects listed in the Methods section. ^#^ *P*-value < 0.10; * *P*-value < 0.05; ** *P*-value < 0.01; *** *P*-value < 0.001.

^2^Hemoglobin is only measured for children 6-59mo in the NFHS.
