# Supplementary material for: Stunting and Wasting Among Indian Preschoolers have Moderate but Significant Associations with the Vegetarian Status of their Mothers
Source: J Nutr. 2020 Mar 14;150(6):1579–89. doi: 10.1093/jn/nxaa042 (PMC7269725; doi:10.1093/jn/nxaa042)
Supplement: nxaa042_Supplemental_Files [file nxaa042_supplemental_files.zip › Online Supplemental Table 9.docx]

**Supplemental Table 9.** Associations between wasting and child consumption of various foods in the last 24h, stratified by child age^1^

|  | Age Range | | | |
| --- | --- | --- | --- | --- |
|  | 6-23mo | 6-11mo | 12-17mo | 18-23mo |
| Dairy | -0.003 (-0.013,0.006) | -0.006 (-0.022,0.009) | 0.01 (-0.007,0.027) | -0.014 (-0.033,0.005) |
| Eggs | 0.014 (-0.011,0.040) | -0.001 (-0.039,0.036) | 0.026 (-0.008,0.061) | 0.011 (-0.028,0.049) |
| Legumes | -0.004 (-0.024,0.016) | 0.022 (-0.015,0.059) | -0.030* (-0.057,-0.003) | 0.003 (-0.026,0.032) |
| Vitamin A rich Fruits or Vegetables | -0.008 (-0.019,0.003) | -0.004 (-0.028,0.020) | 0.001 (-0.016,0.018) | -0.022^#^ (-0.045,0.001) |
| Other Fruits or Vegetables | -0.011 (-0.025,0.002) | 0.001 (-0.025,0.027) | -0.016 (-0.042,0.009) | -0.004 (-0.028,0.021) |
| Flesh Food (Fish, Organ, Meat) | -0.012 (-0.032,0.008) | -0.012 (-0.064,0.041) | -0.002 (-0.034,0.029) | -0.017 (-0.049,0.014) |
| *R*^2^ | 0.044 | 0.063 | 0.065 | 0.079 |
| *n* | 66,562 | 22,417 | 22,134 | 22,011 |

^1^ Table displays the results from adjusted linear probability models of wasting against child consumption from the seven food groups listed in the 24h preceding the survey, adjusting for the control variables and fixed effects listed in the Methods section. Values are βs and 95% confidence intervals based on robust standard errors clustered at the district-level (shown in parentheses). All regressions use the 2015-2016 NFHS data [34] and NFHS weights. ^#^ *P*-value < 0.10; * *P*-value < 0.05; ** *P*-value < 0.01; *** *P*-value < 0.001
